# Efficacy of a physical exercise training programme COPD in primary care: study protocol of a randomized controlled trial

**DOI:** 10.1186/1471-2458-14-788

**Published:** 2014-08-03

**Authors:** Annemieke Fastenau, Jean WM Muris, Rob A de Bie, Erik JM Hendriks, Guus M Asijee, Emmylou Beekman, Rik Gosselink, Onno CP van Schayck

**Affiliations:** Department of Family Medicine, CAPHRI School for Public Health and Primary Care, Maastricht University, PO Box 616, 6200 MD Maastricht, The Netherlands; Department of Epidemiology, CAPHRI School for Public Health and Primary Care, Maastricht University, PO Box 616, 6200 MD Maastricht, The Netherlands; Centre for Evidence Based Physiotherapy, Maastricht University, PO Box 616, 6200 MD Maastricht, The Netherlands; Physical therapy practice, Fysiotherapie Maasstaete, Druten, The Netherlands; Boehringer Ingelheim, Alkmaar, The Netherlands; KU Leuven, Faculty of Kinesiology and Rehabilitation Sciences, Tervuursevest 101, box 1500, 3001 Leuven, Belgium

**Keywords:** Chronic obstructive pulmonary disease, Exercise training, Physical activity, Primary care, Randomized controlled trial

## Abstract

**Background:**

Chronic obstructive pulmonary disease (COPD) is recognized as a systemic illness with significant extra-pulmonary features, such as exercise intolerance and muscle weakness. Pulmonary rehabilitation has been shown to be very effective in counteracting these consequences in patients with more advanced COPD. However, limited data is available on the efficacy of a physical exercise training programme in patients with mild to moderate COPD in primary care. Furthermore, it is unknown if improved exercise capacity translates into enhanced daily physical activities. The aim of this paper is to describe the design of a randomized controlled trial to assess the efficacy of a physical exercise training programme in patients with mild to moderate COPD.

**Methods/design:**

In this randomized controlled trial situated in the primary care setting, 102 patients with mild to moderate airflow obstruction (FEV_1_ ≥ 50% of predicted), dyspnoea and a physically inactive lifestyle will be randomized to an intervention or control group. The intervention group receives a 4-month physical exercise training programme at a local physiotherapy practice, which includes exercise training, resistance training, breathing exercises and advises on how to increase the level of physical activity. The control group receives usual care, i.e. advises on how to increase the level of physical activity and a sham treatment at a local physiotherapy practice of which no physiological training stimulus can be expected. Primary outcome is functional exercise capacity at 4-months measured on the six-minute walk distance. Secondary outcomes include peripheral muscle strength, physical activity in daily life, health related quality of life, Medical Research Council (MRC) dyspnoea score and patients’ perceived effectiveness. Follow-up measurement will take place at 6 months after baseline.

**Discussion:**

This will be one of the first studies to evaluate the efficacy of a physical exercise training programme in patients with mild to moderate COPD completely recruited and assessed in primary care. The results of this trial may give a unique insight into the potential of the implementation of an easy, close-to-home rehabilitation programme.

**Trial registration:**

The Netherlands National Trial Register NTR1471.

## Background

In patients with chronic obstructive pulmonary disease (COPD), disease severity and prognosis are not only determined by lung function impairment, but are also related to extra-pulmonary consequences of COPD such as muscle weakness and exercise intolerance [1–3]. The exercise training component of pulmonary rehabilitation (PR) has been shown to be very effective in improving exercise capacity, dyspnoea and quality of life [4]. As a result, for patients with moderate to very severe COPD and breathlessness (MRC dyspnoea score >2) exercise training is recommended as part of PR in national and international guidelines [1, 5]. Another extra-pulmonary feature of COPD is the decline in daily physical activity (PA) [6]. Only a few trials have investigated the effect of exercise training on changes in daily physical activity [7–11]. These studies showed contradictory results, ranging from significant improvements in PA [8, 9] after exercise training, to small or moderate effects [7, 10] and no effects at all [11]. Overall, current data indicate that exercise training results in a significant but small effect on PA and that larger randomized controlled trials (RCT’s) are needed in this area [12].

A differentiation should be made in the improvement of exercise capacity on the one hand and improvement of daily physical activity on the other. Improvement in exercise capacity fulfils the short-term goal of reducing breathlessness and fatigue [11], improving muscle strength and thus lowers the barriers to be physically more active [10]. Regular physical activity in COPD patients has important long-term health-related benefits, like a lower risk of COPD related hospital admissions and decreased mortality [13]. Furthermore, low physical activity has been associated with systemic inflammation, cardiac dysfunction and lung function decline [13, 14].

All abovementioned studies were performed in a clinical or rehabilitation setting in COPD patients in more advanced GOLD stages [4]. However, patients with moderate COPD also have impairments in exercise capacity, respiratory muscle function, limb muscle force and quality of life [15–18]. In addition, the level of physical activity is already decreased in patients with moderate COPD compared to healthy control subjects [14, 19, 20]. Scant information is available on the effects of community-based exercise training programmes in general and even less about their effect on daily activity [9]. Exercise training programmes in patients with moderate to severe COPD, when incorporated in (self)-management or integrated disease management programmes in primary care, result in improvements in health-related quality of life, breathlessness, exercise capacity, muscle strength, daily physical activity, reduced hospital admissions and hospital days per person [9, 21–24]. Recruitment and assessment was done in the respiratory department of general hospitals [9, 21] or the intervention was multifaceted (i.e. involved more than exercise therapy alone) [22]. To our knowledge hardly any data are available on the efficacy of physical exercise training programmes in patients with mild to moderate COPD that are recruited and treated solely in primary care.

### Relevance

From a patients’ perspective, an increase in exercise capacity and daily physical activity during the early stage of the disease could be beneficial in order to stop the downward spiral of symptom-induced inactivity, deconditioning, muscle weakness, the fear of movement and reduced quality of life. Since behavioural research suggests that modifying behavioural patterns and coping styles takes time to be effective, regular exercise should be started early in the course of the disease for maximal effect [25]. It seems advantageous to initiate exercise training when the symptoms of dyspnoea and deconditioning are not very pronounced. Furthermore, it is proposed that in patients with a relatively preserved lung function, the physiological reserve for improvement is much larger than in patients with (very) severe disease [22].

Treatment of COPD at an early stage could also lessen the burden of disease for society [26]. COPD is one of the leading causes of morbidity and mortality worldwide and imparts a substantial economic burden [27]. COPD-related illness costs are disproportionately distributed, with 10% of the patients (mostly patients in the more severe stages of the disease) accounting for 73% of the total costs, in which hospitalization is the largest contributor [27]. An exercise training programme in primary care will be a relatively cheap and an easily accessible intervention for more patients than an expensive hospital-based rehabilitation programme [28]. Although longitudinal studies are lacking, it is suggested that early recognition of progression of exercise impairment especially in less advanced COPD patients is relevant to prevent further deterioration of functional capacity [22, 29].

### Current daily care for COPD

The Practice Guideline COPD of the Dutch College of General Practitioners (NHG standard, 2007) recommends general practitioners (GP’s) advise all patients with COPD to be sufficient physically active [5]. Referral to a physical exercise training programme in a physiotherapy setting is advised only in patients with moderate to severe COPD, who have impairments in physical functioning due to dyspnoea. As of 2010, there is a new development in the primary care organization of patients with chronic disease in the Netherlands, including COPD. Disease management programmes have regionally been developed by general practitioners in collaboration with other caregivers in primary care. General practitioners and/or the nurse practitioner have a central role. Their COPD care entails lung function testing, prescription of pharmacotherapy and counselling on smoking cessation, inhalation technique and physical activity. There is the possibility to refer patients with mild to moderate COPD to registered physiotherapists, experienced in COPD care. The implementation of this disease management programme for COPD is encouraged by the reimbursement through so-called chained diagnose-treatment combination (DTC) [29]. Although in some regions in the Netherlands these disease management programmes for COPD are already implemented and serve as current daily care, no evidence on the effectiveness of these programmes is available. In addition, no data are available on the added value of a physical exercise training programme compared to advice on increasing daily physical activity in usual care.

### Objectives

The primary objective of our study is to evaluate the *efficacy* of a physical exercise training programme (PETP) in patients with mild to moderate COPD in the primary care setting on exercise capacity, physical activity, dyspnoea and quality of life. The 6 month time point is aimed at gaining more insight into the lasting of the effects.

The secondary objective is to assess how patient characteristics and baseline burden of disease modify the effect of a physical exercise training programme on functional exercise capacity in patients with mild to moderate COPD in primary care.

## Methods/design

### Study design

A randomized controlled trial performed in the primary care setting in which the effects of a 4-month physical exercise training programme for patients with mild to moderate COPD will be compared to a control programme. The latter includes advices concerning physical activity according to the Practice Guidelines of the Dutch College of General Practitioners [5] and a sham-treatment (ST) in physiotherapy practice.

**Figure 1:**
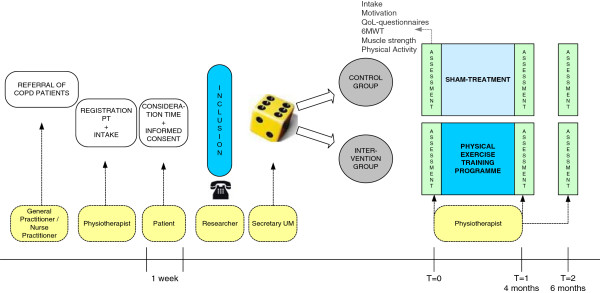
**Overview of the flow of the study.**

General practice is the primary entrance for patients to participate in the trial. If a patient is a potential trial participant, the GP or nurse practitioner explains to the patient that it is important to enhance his physical activity level. For professional support to achieve this goal, the patient is referred to a COPD-certificated physiotherapist. If the patient is willing to undergo the physiotherapy treatment, he/she makes an appointment at the physiotherapy practice. The physiotherapist will give extensive information about the study procedures and the patient will have one week to consider participation in the trial. If the patient wants to participate, randomization will take place after obtaining informed consent. Patients of both treatment groups will be assessed in physiotherapy practice at baseline, at the end of the programme (after four months) and six months after baseline assessment. The measurements done by the physiotherapist are part of their routine assessments and are imbedded in the intake procedures. The results of this intake procedure will be the starting point of the treatment. Figure 1 illustrates the flow of the study.

The ethics committee of Maastricht University has approved the study protocol, procedures and informed consent and the trial has been registered at The Netherlands National Trial Register NTR1471.

### Setting

This multicenter trial is coordinated by the CAPHRI School for Public Health and Primary Care of Maastricht University and conducted in several general practices and physiotherapy practices in the southern part of the Netherlands.

### Study population

Participants for the trial will be recruited from general practices in Limburg, in the southern part of the Netherlands. There are 614 general practices in this region covering a population of over 1.000.000 [30]. Statistics suggest that 1,7% of the Dutch population is likely to have COPD [31]. The population in Limburg is the least physically active population of the Netherlands [32].

Inclusion will be based on patients with a clinical diagnosis of mild to moderate COPD (post-bronchodilator FEV_1_/FVC ratio < 0,7 and FEV_1_ ≥ 50% of predicted); who not have a minimum of 30 minutes of physical activity at moderate intensity, on at least 5 days per week, according to the ACSM-recommendation [33]; having a stable situation (no exacerbations in the last 8 weeks) and adequate and optimal inhalation technique, are competent enough to understand and speak the Dutch language and having provided written informed consent. According to the Practice Guidelines of the Dutch College of General Practitioners, a maximal cardiopulmonary exercise test prior to exercise training will be conducted by a pulmonologist, cardiologist or a sports medicine physician in COPD patients who suffer from cardiopulmonary co morbidities [5, 34].

Patients will be excluded from the trial, when they: already receive or have received a physical exercise training programme or rehabilitation therapy in the past year; have had respiratory tract infections within the last 8 weeks; are suffering from serious co morbid conditions, which would interfere with regular exercise training (including severe orthopaedic, muscular, neurological disorders or cardiovascular conditions) and patients who are expected to be lost for follow-up (e.g. because of a planned change of residency or a long holiday break).

### Randomization and blinding

After informed consent is obtained, patients will be randomly assigned to the intervention group or the control group in a 1:1 ratio, with the help of computer generated random number tables. Randomization will be performed by a centralized and independent person who has a concealed list. The researcher is not involved in allocation to treatment group or setting. Full blinding procedures are not applicable in this study. The participating general practitioners, nurse practitioners and physiotherapists and also the patients cannot be blinded to allocation of individuals to the intervention group or control group. Although patients will be aware of the existence of two treatment arms, they are not informed about the exact content of the other treatment arm. Also, patients of both groups will not be in the same physiotherapy setting for treatment at the same time. The researcher will be fully blinded to the randomization list until the clinical database is unlocked at the end of the trial.

### Sample size calculation

We performed a pilot study in one physiotherapy setting in order to get a rough idea of the improvement in six-minute walk distance (6MWD) in this group of patients after a 4-month physical exercise training programme. Data of six patients with mild to moderate COPD were collected. Their mean 6MWD was 475 metres with a standard deviation of 62. These patients increased their 6MWD by a mean of 52 metres. Troosters et al. reported a mean difference in change from baseline of 52 metres between the intervention group and control group, in a study of the short- and longer-term benefits of 6 months pulmonary rehabilitation [35]. A more recent study showed an improvement of 54 metres with a standard deviation of 78 metres after a 7 week pulmonary rehabilitation programme [11]. Our assumptions for this study are that the mean difference in change from baseline between the intervention group and the control group (δ) is 52 metres (52–0 m), with a standard deviation (σ) of 78 metres [11]. According to the effect size measures for two independent groups this implies a large effect size (Cohen’s d = δ/σ = 52/78 = 0.66) [36]. The sample size needs to be 36 patients per treatment arm (72 in total), whereby α = 0.05 (2 tailed), 80% power and a ratio of control to experimental patients of 1:1. This sample size calculation is done by using the PS power and sample size program of Dupont and Plummer [37, 38].

A total patient number of 102 participants (51 per treatment arm) is needed, based on the abovementioned calculation and allowing for dropouts (30%). Previous studies showed a drop-out rate varying from 26 to 31% [11, 35, 39, 40].

### Intervention

The treatment period is set at four months and consists of either the physical exercise training programme or the sham-treatment. The intervention will be carried out by physiotherapists working in a primary care setting, with broad expertise and education in COPD. The treatment protocol is standardised, the Dutch Guideline Physiotherapy in COPD serves as a framework, and based on individual problems and possibilities of the patient [41].

#### Intervention group (Physical Exercise Training Programme)

The physical exercise training programme has four main goals; improvement in exercise capacity, muscle strength, daily physical activity and breathing technique. The physical training component entails endurance training and/or interval training [41]. Walking speed on the treadmill is executed with an intensity of 75% or higher of the results of the six-minute walk test (6MWT). Ratings of perceived exertion and dyspnoea of five and higher on the modified Borg-scale (0–10) are used to tailor exercise intensity [42].

Resistance training in addition to endurance or interval training is recommended in all patients, especially important in patients with peripheral muscle weakness. In the absence of any comparative studies it is recommended to use both upper limb and lower limb resistance weight training at an intensity of at least 60-80% of the one repetition maximum, 2 to 3 sets of 8–12 repetitions are preferable [41].

Much emphasis is given to the assessment and treatment of physical inactivity in daily life. Patients are advised to increase their total physical activity. Patient and physiotherapist together define a strategy to meet the ACSM-recommendation for physical activity, i.e. performing moderately intense physical activity for 30 minutes on at least five days a week [33].

Breathing exercise is an embracing term for a range of exercises such as active expiration, slow and deep breathing, pursed lips breathing, relaxation therapy, body positions such as forward leaning and diaphragmatic breathing [41].

The physical exercise training programme will consist of two supervised sessions per week in the physiotherapy setting in primary care. These sessions will be with 1–5 patients at the time and the duration of each session will be 60–90 minutes, depending on group size. From an organisational and practical point of view it is not feasible to ask patients to come to a physical therapy setting more often. Furthermore, as mentioned before, an important part of the programme is enhancement of daily physical activity. So, patients are requested to perform an additional training session at home, including walking and/or cycling and they have to report these activities weekly to the physiotherapist. It is our aim to enhance the awareness and responsibility of our patients to change their physical activity behaviour for the long term and this encompasses enhancement of self-management and self-efficacy.

#### Control group (sham-treatment)

According to the national guidelines of the Dutch College of General Practitioners (NHG) and the Multidisciplinary Guideline on non-pharmacological treatment of COPD, the GP and the nurse practitioner should give advice to improve the physical condition [5, 43]. Verbal advice will be supported by a written brochure. This brochure is developed in collaboration with the NHG as part of a preceding implementation project of physiotherapy for COPD patients in primary care [44].

In addition, the patients in the control group will participate in a sham-treatment at the physiotherapy practice. This treatment consists of 30 minutes once a week “exercise” training, with ratings of perceived exertion and dyspnoea of 2 or lower on a modified Borg-scale. It is unlikely that a physiologic training stimulus will occur at these levels of exertion. There will be no breathing exercises or resistance training. Furthermore, patients will be advised to do at least 30 minutes of moderate intense physical activities on at least five days a week according to the ACSM-recommendation for physical activity [33].

### Outcome measures

All outcomes will be assessed at baseline (T0), at the end of intervention after four months (T1) and at the end of follow-up (T2) at six months.

#### Primary outcome measure

The primary outcome measure will be the functional exercise capacity measured by the increase in the six-minute walk distance (6MWD) at 4 months compared to baseline. The six-minute walk test (6MWT) will be performed in accordance with the ATS Statement: guidelines for the 6MWT [45], except that a standard 30-meter corridor will not always be feasible in a primary care physiotherapy practice, but the minimal track will be 10 meter. The results will be expressed in absolute values and as percent of the predicted value [46]. During the walk test, perceived fatigue and dyspnoea will be measured on a modified Borg scale ranging from zero (nothing at all) to ten (very, very severe) [47]. Oxygen saturation and pulse rate will be measured by a finger pulse oximeter (Onyx 9500) [45].

#### Secondary outcome measures

Isometric handgrip force will be measured with a hydraulic handheld dynamometer (Yamar Preston, Jackson MI). Peak handgrip force (in Newton) will be assessed at the dominant side with the elbow at 90 degrees flexion, with the underarm and wrist in neutral position [48]. Isometric knee extension and shoulder abduction force will be measured in standardised positions by a handheld dynamometer by means of the break method [42, 49]. Peak torques will be measured at the dominant side according to Andrews et al. [49]. At least three attempts will be performed for all muscle tests.

Self-reported daily physical activity will be assessed by the brief physical activity assessment tool [50]. Objective daily physical activity will be measured during 3 consecutive days and nights with an accelerometer-based activity monitor (Dynaport; McRoberts BV). Data of both intensity of movement and duration will be collected, like steps per day, total active time per day, time spent in moderate intense physical activities and vigorous activities and physical activity level (PAL). All patients will be carefully instructed on how the activity monitor should be positioned and they will receive a manual with clear instructions and figures. They will also have to fill out a checklist to verify if their day was a representative one and to indicate any possible hindrance of the activity monitor.

The level of dyspnoea will be assessed by the Medical Research Council (MRC) dyspnoea score [51]. Specific Health Related Quality of Life (HRQL) will be assessed by means of the Clinical COPD Questionnaire (CCQ) [52, 53] and the Chronic Respiratory Questionnaire (CRQ-SR) [54–56]. The global perceived effect (GPE) of the treatment according to the patients will be measured on a GPE scale [57, 58].

Furthermore, the following baseline characteristics will be measured, height, weight, Body Mass Index (BMI) and level of motivation by means of the questionnaire (Dutch translation) according to Miller and Rollnick et al. [59–61].

### Data analysis

The descriptive characteristics will be presented quantitatively as means (±standard deviation) for continuous variables and as medians for categorical variables and will be presented for the total group, as well as for the separate groups. Unpaired t-tests will be used to compare the effects of the treatment between the intervention group and control group at the end of the physical exercise training programme (4 months). P-values smaller than 0.05 will be considered as statistically significant.

Group (intervention vs. control) by time (pretest vs. posttests) repeated measurements analysis of variance (RM ANOVA) will be performed to examine (1) intervention main effect, (2) time main effect, and (3) intervention by time interaction effect on each of the continuous primary and secondary outcomes. An analysis of covariance will be done to evaluate the relationship between covariates and the dependent variable. The random effect will also be evaluated, since participants from both arms will be nested within the same physiotherapy setting. To evaluate which factors predict a positive outcome, i.e. the physical exercise training programme is effective, a multiple linear regression analysis will be done, using interaction terms between predictors and physical exercise. A predictor variable will make a significant contribution to predicting the outcome when P-value is smaller than 0.05. The following possible predictors are taken into account, baseline: MRC dyspnoea score, walking distance, peripheral muscle strength, level of daily physical activities and compliance with the training programme [62–64].

## Discussion

This will be one of the first studies to evaluate the efficacy of a physical exercise training programme in patients with mild to moderate COPD completely recruited and assessed in primary care. If the results of this study show that this training programme is effective, this would mean a big step ahead in the follow-up of patients with mild to moderate COPD. Patients are actively involved in their disease management in an early phase and the intervention can ameliorate further deterioration and influence their prognosis in the long term [22]. Exercise training has been shown to positively affect some aspects of health status (exercise capacity, muscle force, blood pressure, bone mass) [65]. Using an active lifestyle is necessary to break out of the negative spiral of dyspnoea and deconditioning and is probably essential for a long lasting change in improvements in daily physical activity. The availability of a physical exercise training programme close to the patient’s home most likely improves compliance to the enhanced physical activity [22]. With this enhanced physical activity and the benefits of an improved exercise capacity, a patient can regain his social contacts [22]. This will give a great impact of a patient’s quality of life.

### Bias, confounders and limitations

From a methodological point of view, a cluster randomized design would be the most sound design for the study. The rationale for performing an individually randomized trial is that we observed in a pilot study [44], that physiotherapists were not very keen to deliver a treatment without a proper training programme, although hard evidence of efficacy of a physical exercise training programme COPD was still lacking. Therefore, it would be impossible to recruit sufficient physiotherapy practices with treating *only* control patients. We have found a solution to this problem by allowing the physiotherapist offering the possibility for patients in the control group to participate in the physical exercise training programme after the study-period, in case the intervention has proven to be efficacious. To tackle contamination, we will train and instruct the physiotherapists thoroughly in advance of the study and monitor and instruct them throughout the intervention period. Physiotherapists can only participate if they are willing to deliver both the intervention treatment and sham-treatment. Another strategy to minimize contamination is that patients of the intervention and control group will not be in the same physiotherapy setting at the same time. So, the physiotherapists can focus their mind on just one treatment at the time.

Both the physiotherapists and the patients are not blinded during this study, since they are aware of the treatment procedures. Physiotherapists will conduct the measurements as well as the treatment in patients. Due to practical considerations it is not feasible to perform all measurements in many different practice settings by a single researcher. To assure a high quality and univocal treatment, the participating physiotherapists will be trained and instructed extensively before the start of the training. Also, throughout the intervention period the physiotherapists will be monitored continuously. The researcher will visit the participating physiotherapy practices frequently and will have regular contact by telephone and email in order to check the compliance with the treatment protocols.

As the population in Limburg is the least physically active population of the Netherlands, this might influence the external validity of the study. Another limitation is that the six-minute walk tests are performed on different tracks, which will influence the variability. An advantage of the randomization on patient level instead of physiotherapy practice level is that patients are assigned to smaller and longer passages in a non-differential manner and an equal distribution of patients from the intervention group and control group can be expected per physiotherapy practice. Since we are interested in the difference scores (4 or 6 months minus baseline measurement) and participants are assessed in the same passage on all occasions, we think that the variability is acceptable.

### Potential barriers

From the feasibility study of Faulkner et al. it is known that recruitment of patients with moderate COPD for a physical activity intervention in primary care is very difficult [40]. The reported main recruitment issue for caregivers in that study was lack of available time to participate in research activities [40]. Furthermore, in general practice no objective tool to measure daily physical activity is available, only subjective questionnaires. As a consequence, general practitioners or practice nurses might have a lack of information on this topic and will not consider a follow-up strategy, including referral to a physical exercise training programme.

One of the first major symptoms in COPD is exertional breathlessness. To avoid confrontation with this symptom, patients with COPD are more inclined to adapt their lifestyle, for example taking the elevator instead of climbing the stairs. In this way impairments in daily life are not noticed by the patient. So, on patients’ level it might be a barrier that patients with only a mild airway obstruction and moderate exercise limitation do not feel the need to participate in a physical exercise training programme [66]. In this mild to moderate category, many patients will probably have work commitments and lack of time might be a problem [40].

A key factor in the recruitment of patients is the teamwork between the different healthcare professionals in primary care. From June 2006 till November 2007, our research group has executed an implementation project of physiotherapy for COPD patients in primary care in the region to be studied [44]. One of the main objectives was to start up and improve collaboration between general practitioners, nurse practitioners and physiotherapists. As a result of the project referral policy of COPD patients to physiotherapists in primary care in this region improved. In this way, we expect to minimize recruitment problems.

Given the evidence of the efficacy of pulmonary rehabilitation on functional exercise capacity, dyspnoea and quality of life in patients with moderate to severe COPD, there is now an urgent need to determine whether similar observations apply in the larger group of patients with earlier disease characteristics [40].

## Abbreviations

BMI: Body Mass Index
CCQ: Clinical COPD Questionnaire
COPD: Chronic obstructive pulmonary disease
CRQ: Chronic Respiratory Questionnaire
DTC: Diagnose-treatment combination
FEV_1_: Forced expiratory volume in one second
FVC: Forced vital capacity
GOLD: the Global Initiative for Chronic Obstructive Lung Disease
GP: General practitioner
GPE: Global perceived effect
HRQL: Health Related Quality of Life
MRC: Medical Research Council
NHG: Dutch College of General Practitioners
PAL: Physical activity level
PETP: Physical exercise training programme
PR: Pulmonary rehabilitation
RCT: Randomized controlled trial
ST: Sham-treatment
6MWD: Six-minute walk distance
6MWT: Six-minute walk test.
